# Cancer‐specific survival after diagnosis in men versus women: A pan‐cancer analysis

**DOI:** 10.1002/mco2.145

**Published:** 2022-06-30

**Authors:** Yan He, Yonglin Su, Junsong Zeng, Weelic Chong, Xiaolin Hu, Yu Zhang, Xingchen Peng

**Affiliations:** ^1^ Department of Biotherapy, West China Hospital Sichuan University Chengdu China; ^2^ Department of Rehabilitation West China Hospital Sichuan University Chengdu China; ^3^ Department of Medical Oncology Thomas Jefferson University Philadelphia Pennsylvania USA; ^4^ Department of Nursing, West China Hospital Sichuan University Chengdu China; ^5^ Affiliated Hospital of Chengdu University Chengdu China

**Keywords:** cancer, gender differences, prognosis, risk factor, survival

## Abstract

Comprehensive understanding of cancer‐specific survival differences in gender is critical for cancer prevention and treatment. Based on the Surveillance Epidemiology and End Results database, we included data from the most prevalent cancers (lung, esophageal, liver, pancreatic, stomach, colorectal, kidney, and bladder cancer). Cox proportional hazards regression models were constructed to estimate hazard ratios, simultaneously adjusting for demographic, clinical, and treatment factors. Overall, male patients had a worse cancer‐specific survival than female patients. After adjustment for cancer prevalence with 1:1 matching, gender remained a significant factor in cancer‐specific survival. Among the included cancer types, female patients showed survival benefit in lung, liver, colorectal, pancreatic, stomach, and esophageal cancer, and male patients showed better survival in bladder cancer. Except for kidney cancer, the gender disparity was consistent between cancer patients with nonmetastatic and metastatic disease. Overall, gender appears to be a significant factor influencing cancer‐specific survival, and the prognosis of female patients is better than male patients in most cancers. This work might inspire the development of strategies for gender‐specific precision cancer prevention and treatment.

## Graphical abstract

Comprehensive understanding of survival differences in gender after cancer diagnosis is critical for cancer prevention and treatment. We compared the gender differences of cancer‐specific survival in lung, esophageal, liver, pancreatic, stomach, colorectal, kidney, and bladder cancer. Totally, gender seemed to be a significant factor influencing cancer‐specific survival, and the prognosis of female patients was better than male patients.

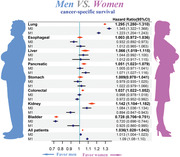

## 1 INTRODUCTION

As a major public health problem, cancer is one of the leading causes of death in the world.^1^ According to the GLOBOCAN 2020 estimates of cancer incidence and mortality, the global burden of cancer worldwide estimated 19.3 million new cancer cases and 10.0 million cancer deaths.^2^ Among them, the new cancer cases and deaths of males (10.1 million and 5.5 million) are higher than females (9.2 million and 4.4 million).^2^ Thus, gender may be a significant factor influencing cancer incidence and mortality.

Gender differences are reflected in several aspects of life, such as hormone levels, behavioral psychology, economic preferences, emotional characteristics, physical strength, and immune system.^3^, ^4^ Sex hormones regulate the expression and function of multiple signaling pathway and are important in mediating apoptosis, autophagy, and immune function.^5^ Previous studies reported that gender plays an important role in cancer‐specific survival and drug response. It has been observed that women could obtain better cancer‐specific survival in breast and colorectal cancer, when compared with male patients.^6^, ^7^ Furthermore, 2‐methoxyestradiol, which is the physiological estrogen metabolite, could be considered as a promising drug against colorectal,^8^ pancreatic,^9^ and liver cancer.^10^ Male cancer patients tended to derive a larger relative benefit from immunotherapy than female patients.^11^ Considering the complex relationship between gender and cancer, it is important to systematically understand the association of gender difference with cancer prognosis.

The findings of cancer‐specific survival differences between male and female cancer patients were controversial. In bladder cancer, two prior studies identified that gender is an independent risk factor for reduced overall survival.^12^, ^13^ However, recently, a cohort study observed that gender cannot be used to predict outcomes, and the 5‐year cancer‐specific survival for male patients was 66.2% when compared to 66.6% for female patients (*p* = 0.55).^14^ In colorectal cancer, it was shown that female patients had a significantly longer overall survival than male patients (hazard ratios (HR) 0.78, 95% CI 0.77–0.80),^15^ but a meta‐analysis observed that male patients had a better prognosis than female patients (overall survival, HR 0.82, 95% CI 0.68–0.99, *p* = 0.039).^16^ In stomach cancer, it was reported that female patients presented with earlier stage, lower grade, and significant better prognosis than male patients.^17^ However, another study found that female patients had a higher rate of relapse risk than male patients in stomach cancer (risk ratio 2.47, 95% CI 1.04–5.89).^18^ Thus, whether gender is a significant factor influencing cancer prognosis remains unclear.

To address this unresolved issue, we provide an overview of gender differences in cancer prognosis from the Surveillance Epidemiology and End Results (SEER) database, which is a large‐scale population‐based database from 18 states of the USA.^19^ Firstly, gender disparity of cancer prognosis was compared in all patients, and further stratified according to primary cancer type. Then, patients were divided into subgroup by nonmetastatic (M0) and metastatic status (M1). Finally, demographic, clinical, and treatment factors were added sequentially into adjustment models to reduce influence by confounding factors. Overall, cancer‐specific survival of female cancer patients was better than male. Moreover, female cancer patients showed survival benefit in lung, liver, pancreatic, colorectal, stomach, and esophageal cancer after adjustment. Male patients only showed better survival in bladder cancer. Except for kidney cancer, gender disparity in cancer prognosis was consistent between M0 and M1 disease. In summary, gender seems to be a significant factor influencing cancer‐specific survival, and the prognosis of female patients is better than male patients. This work might inspire the development of strategies for clinical trials and precision treatment that better targets certain gender demographics.

## 2 RESULTS

### 2.1 The characteristics of patients

**FIGURE 1:**
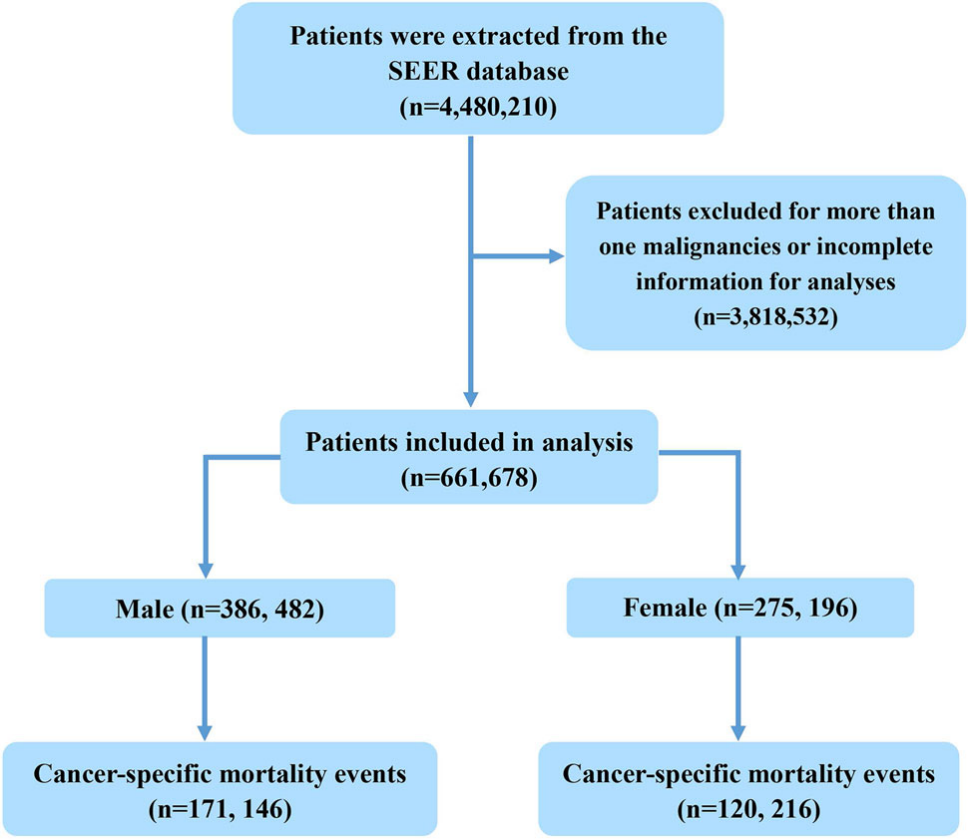
TRIPOD flow Diagram

A total of 661,678 patients were eligible for analysis. Among them, 386,482 cases were male, and 275,196 were female (Figure 1). There were 503,568 patients (76.10%) with nonmetastatic (M0) disease and 158,110 patients (23.90%) with metastatic (M1) disease at diagnosis. This study included patients across all ages. The median age of total patients was 67 years (95% CI, 57–76 years), and median age of each cancer type is shown in Table 1.

**TABLE 1:** Summary characteristics of patients

				5‐year cancer‐specific survival rates (95% CI)	
Valuables	Number of patients	Male ratio	Age median (IQR), years	Female	Male
Total patients	661,678	58.41%	67 (57–76)	53.4% (53.2%–53.6%)	52.5% (52.3%–52.6%)
M0 disease					
Lung cancer	101,098	52.1%	69 (61–76)	47.7% (47.2%–48.2%)	37.6% (37.1%–38.0%)
Esophageal cancer	19,952	78.8%	66 (58–75)	31.4% (29.8%–33.0%)	32.0% (31.1%–32.8%)
Liver cancer	13,893	75.3%	62 (56–71)	38.7% (36.8%–40.6%)	36.3% (35.2%–37.4%)
Pancreatic cancer	16,793	50.3%	66 (58–75)	19.6% (18.6%–20.6%)	18.8% (17.8%–19.8%)
Stomach cancer	14,340	56.0%	70 (58–79)	43.5% (42.2%–44.9%)	43.8% (42.6%–45.0%)
Colorectal cancer	184,319	50.7%	67 (56–77)	78.7% (78.4%–79.0%)	78.7% (78.4%–79.0%)
Kidney cancer	66,630	61.3%	61 (52–70)	88.2% (87.8%–88.7%)	87.7% (87.3%–88.0%)
Bladder cancer	86,543	74.3%	70 (61–79)	76.2% (75.6%–76.8%)	81.9% (81.6%–82.2%)
M1 disease					
Lung cancer	67,652	55.8%	67 (59–75)	5.9% (5.6%–6.2%)	3.6% (3.4%–3.9%)
Esophageal cancer	12,271	82.1%	64 (56–72)	4.2% (3.3%–5.4%)	2.9% (2.6%–3.4%)
Liver cancer	2660	53.8%	62 (55–72)	3.0% (1.6%–5.6%)	2.5% (1.8%–3.5%)
Pancreatic cancer	9648	53.5%	66 (57–75)	4.3% (3.7%–5.0%)	3.0% (2.5%–3.6%)
Stomach cancer	9508	57.7%	64 (53–75)	2.5% (2.0%–3.2%)	3.0% (2.5%–3.6%)
Colorectal cancer	44,814	78.6%	63 (54–74)	13.0% (12.5%–13.5%)	12.1% (11.6%–12.6%)
Kidney cancer	7642	67.5%	62 (54–70)	14.2% (12.7%–15.9%)	16.7% (15.5%–17.9%)
Bladder cancer	3915	69.2%	70 (60–79)	5.3% (4.0%–7.1%)	4.7% (3.8%–5.9%)

Abbreviations: IQR, interquartile range; M0, nonmetastatic; M1, metastatic; 95% CI, 95% confidence interval.

### 2.2 Cancer‐specific survival in men versus women in all patients

**FIGURE 2:**
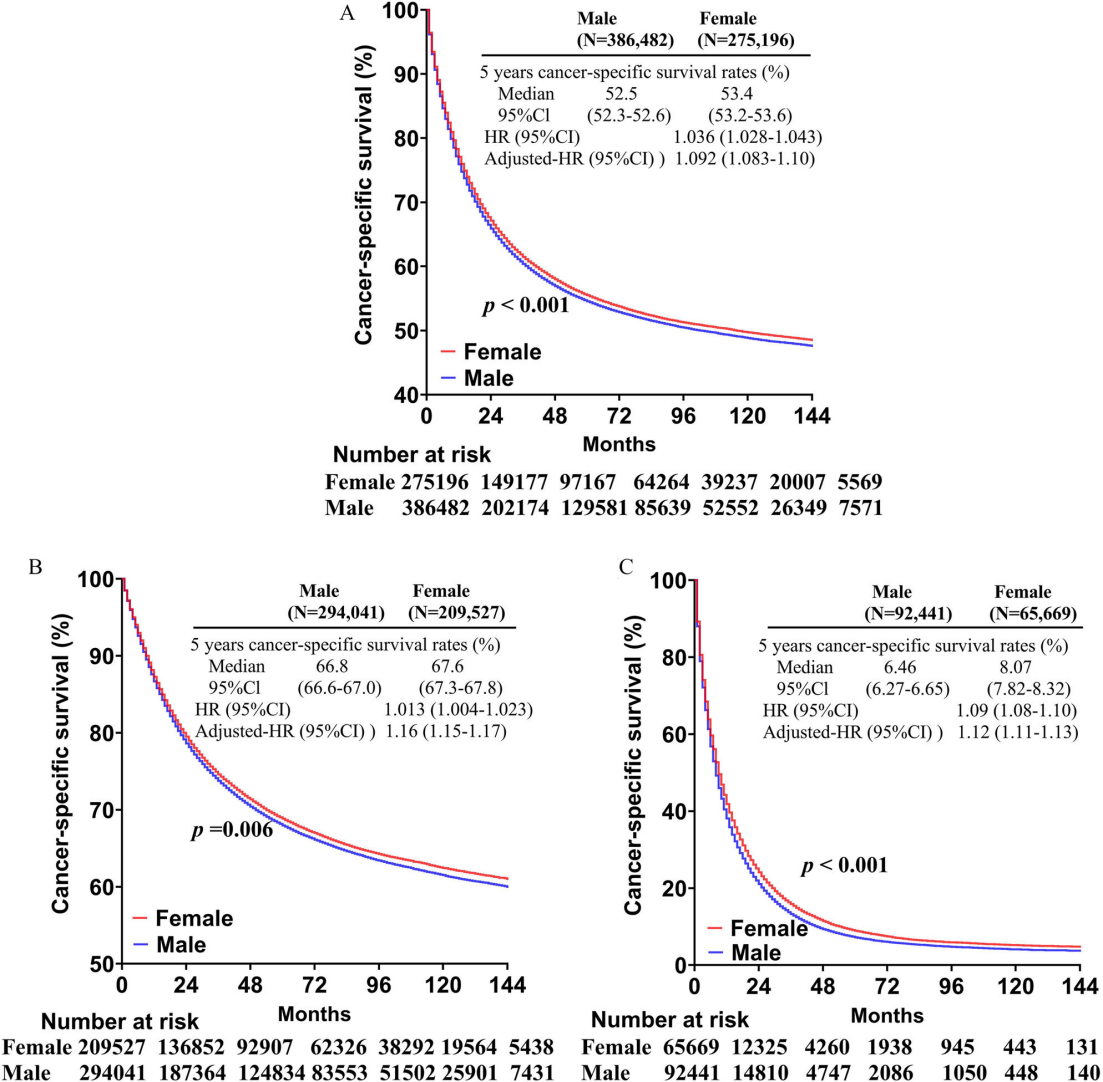
Cancer‐specific survival of men versus women in all patients (A), nonmetastatic, (B) and metastatic diseases (C)

Gender disparity of cancer prognosis was analyzed in all eligible patients. Overall, the results showed that male had worse cancer‐specific survival (CSS) (HR 1.036, 95% CI 1.028–1.043), compared with their female counterparts. The 5‐year CSS rate in M0 disease was 66.8% (95% CI 66.6%–67.0%) in men versus 67.6% (67.3%–67.8%) in women (HR 1.013, 95% CI 1.004–1.023, *p* = 0.006). The 5‐year CSS rates in M1 disease were 6.46% (6.27%–6.65%) in men versus 8.07% (7.82%–8.32%) in women (HR 1.09, 95% CI 1.08–1.10, *p* < 0.001) (Figure 2).

### 2.3 Cancer‐specific survival in men versus women when stratified by primary cancer type

**FIGURE 3:**
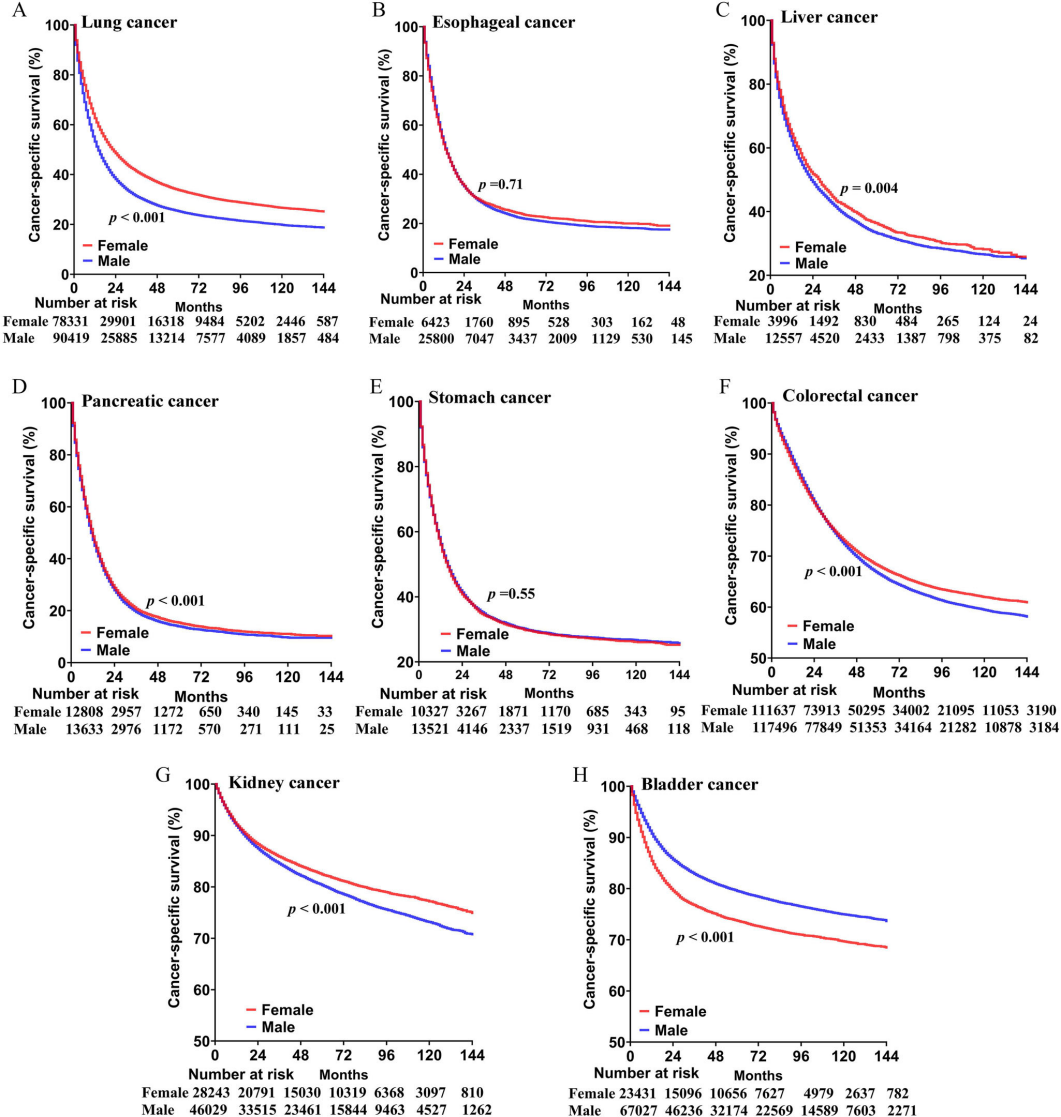
Cancer‐specific survival of men versus women among lung (A), esophageal (B), liver (C), pancreatic (D), stomach (E), colorectal (F), kidney (G), and bladder cancer (H)

**FIGURE 4:**
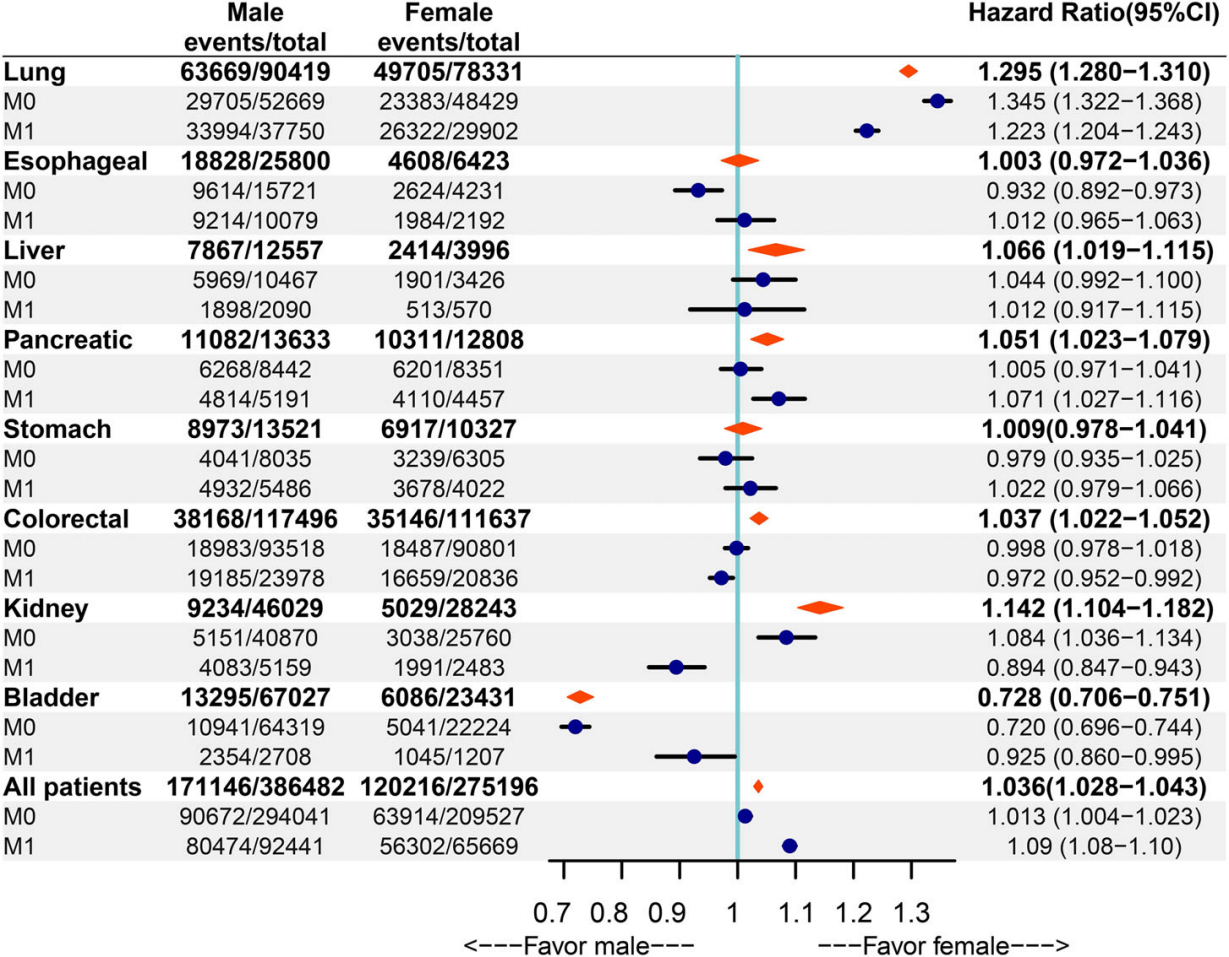
Forest plot of cancer‐specific survival in men versus women

When stratified by the type of primary cancer, women showed survival benefit in lung (HR 1.295, 95% CI 1.28–1.31), liver (HR 1.066, 95% CI 1.019–1.115), colorectal (HR 1.037, 95% CI 1.022–1.052), pancreatic cancer (HR 1.051, 95% CI 1.023–1.079), and kidney (HR 1.142, 95% CI 1.104–1.182). Conversely, male patients appear to have better survival in bladder cancer (HR 0.728, 95% CI 0.706–0.751). In addition, the disparity was insignificant in stomach (HR 1.009, 95% CI 0.978–1.041) and esophagus cancer (HR 1.003, 95% CI 0.972–1.036) (Figures 3, 4).

### 2.4 Subgroup analysis of cancer‐specific survival in M0 and M1 disease

Subgroup analysis was conducted in patients with M0 and M1 disease. The subgroup analyses of the M0 disease showed that female patients had better CSS after the diagnosis of lung cancer (HR 1.345, 95% CI 1.322−1.368, *p* < 0.001) and kidney cancer (HR 1.084, 95% CI 1.036−1.134, *p* < 0.001). There were two cancers presenting survival benefit in male patients including esophageal cancer (HR 0.932, 95% CI 0.892−0.973, *p* = 0.001) and bladder cancer (HR 0.72, 95% CI 0.696−0.744, *p* < 0.001) in M0 disease. The differences were insignificant in liver cancer (HR 1.044, 95% CI 0.992−1.1, *p* = 0.1), pancreatic cancer (HR 1.005, 95% CI 0.971−1.041, *p* = 0.77), stomach cancer (HR 0.979, 95% CI 0.935−1.025, *p* = 0.36), and colorectal cancer (HR 0.998, 95% CI 0.978−1.018, *p* = 0.85) between men and women (Figures S1 and S2).

As for patients of M1 disease, two cancers presented a survival benefit in women, including lung cancer (HR 1.223, 95% CI 1.204−1.243, *p* < 0.001) and pancreatic cancer (HR 1.071, 95% CI 1.027−1.116, *p* = 0.001). In M1 disease, three cancers presented a survival benefit in men: colorectal cancer (HR 0.972, 95% CI 0.952−0.992, *p* = 0.008), kidney cancer (HR 0.894, 95% CI 0.847−0.943, *p* < 0.001), and bladder cancer (HR 0.925, 95% CI 0.86−0.995, *p* = 0.039). The other cancers had insignificant difference between men and women, such as esophageal cancer (HR 1.012, 95% CI 0.965−1.063, *p* = 0.59), liver cancer (HR 1.012, 95% CI 0.917−1.115, *p* = 0.79), and stomach cancer (HR 1.022, 95% CI 0.979−1.066, *p* = 0.35) (Figures S3 and S4).

### 2.5 Cancer‐specific survival after adjustment for associated factors in men versus women

Demographic, clinical, and treatment factors were added sequentially to establish adjustment models to reduce potential bias caused by confounding factors. As shown in Table 2, the results in model 1 (adjusted for the calendar year of diagnosis) were similar to unadjusted models in all cancers, which indicated that screening or treatment changes over decades were not a factor associated with the gender disparity in CSS. After adjusting for year of diagnosis, demographic, clinical, and treatment factors (model 4), gender remained a significant factor in CSS, with women having a better CSS overall (HR 1.092, 95% CI 1.083–1.1).

**TABLE 2:** Gender differences in prognosis after adjustment

Valuables		Cancer‐specific survival, male versus female, HR (95% CI)			
	Unadjusted‐model	Model 1	Model 2	Model 3	Model 4
Total patients	1.036 (1.028–1.043)	1.036 (1.029–1.044)	1.088 (1.080–1.096)	1.144 (1.135–1.152)	1.092 (1.083–1.100)
Lung cancer	1.295 (1.280–1.310)	1.290 (1.275–1.306)	1.338 (1.321–1.354)	1.253 (1.238–1.269)	1.236 (1.221–1.251)
Esophageal cancer	1.003 (0.972–1.036)	1.006 (0.974–1.039)	1.080 (1.045–1.116)	1.066 (1.031–1.103)	1.130 (1.093–1.169)
Liver cancer	1.066 (1.019–1.115)	1.070 (1.022–1.120)	1.121 (1.069–1.174)	1.115 (1.064–1.169)	1.083 (1.034–1.135)
Pancreatic cancer	1.051 (1.023–1.079)	1.056 (1.028–1.085)	1.107 (1.077–1.138)	1.070 (1.041–1.100)	1.067 (1.038–1.097)
Stomach cancer	1.009(0.978–1.041)	1.011 (0.979–1.043)	1.050 (1.017–1.085)	1.060 (1.026–1.095)	1.049 (1.015–1.083)
Colorectal cancer	1.037 (1.022–1.052)	1.038 (1.023–1.053)	1.123 (1.106–1.140)	1.099 (1.082–1.115)	1.083 (1.067–1.100)
Kidney cancer	1.142 (1.104–1.182)	1.150 (1.111–1.190)	1.224 (1.181–1.268)	0.986 (0.952–1.022)	0.999 (0.964–1.035)
Bladder cancer	0.728 (0.706–0.751)	0.728 (0.706–0.750)	0.845 (0.818–0.872)	0.921 (0.891–0.950)	0.910 (0.881–0.940)

*Note*: Model 1 was adjusted for the year of diagnosis. Model 2 was adjusted for the year of diagnosis age, race, and marital status of at diagnosis. Model 3 was adjusted for the year of diagnosis, age, race, marital status of at diagnosis, histologic type, histologic grade, and clinical stage. Model 4 was adjusted for the year of diagnosis, age, race, marital status of at diagnosis, histologic type, histologic grade, clinical stage, and patients whether accepted chemotherapy, radiotherapy, and surgery.

Abbreviation: HR, hazard ratios; 95% CI, 95% confidence interval.

Significant difference of CSS between men and women persisted after accounting for demographic, clinical, and treatment factors in the following cancers. In lung (HR 1.236, 95% CI 1.221–1.251), liver (HR 1.083, 95% CI 1.034–1.135), pancreatic (HR 1.067, 95% CI 1.038–1.097), and colorectal cancer (HR 1.083, 95% CI 1.067–1.1), the CSS of women was significantly higher than males. In contrast, men had a better CSS in bladder cancer (HR 0.91, 95% CI 0.881–0.94). Additionally, gender was an insignificant factor in the unadjusted model for esophageal and stomach cancer, but the CSS of women was significantly higher than men when demographic, clinical, and treatment factors were accounted. The only exception to this observation is kidney cancer, where a female‐favored CSS (HR 1.142, 95% CI 1.104−1.182) became nonsignificant after adjustment (HR 0.999, 95% CI 0.964–1.035).

Moreover, in order to reduce potential bias caused by confounding factors, propensity‐score matching analysis (a ratio of 1:1) was used. After propensity score matching, gender still appeared to be a significant factor influencing cancer‐specific survival, and the cancer prognosis of female patients was better than that in male patients in total patients (Table S1).

## 3 DISCUSSION

In this study, we searched large‐scale data from SEER database to provide an overview of gender differences in cancer prognosis and found that female patients had a better cancer‐specific survival compared with male patients. These advantages in female patients also existed in most of the cancers after adjustment, such as lung, liver, pancreatic, colorectal, stomach, and esophageal cancer. Only bladder cancer presented survival benefit in male patients. Furthermore, in esophageal and stomach cancer, from unadjusted model to adjusted model, the survival benefit in female became more and more conspicuous.

Several evidence supported that women had a significant survival benefit after cancer diagnosis. Smoking is more prevalent in males, and most male patients with lung cancers are attributed to smoking.^20^ Meanwhile, current smokers at cancer diagnosis tend to have poorer prognosis (5‐year overall survival was 31.2% versus 42.4% in smokers versus never‐smokers).^21^ In addition, several biological speculations are associated with better cancer prognosis in women, such as gene expression, hormonal regulation, immune function, oxidative damage, and autophagy.^22^ It was reported that genes on the X chromosome regulated immune function. In detail, immunoglobulin concentrations, CD4+T cell numbers, CD4/CD8 T cell ratios, and B cell numbers of patients with Klinefelter syndrome (XXY syndrome) were higher than XY male.^23^ Meanwhile, another study was also observed that female had higher basal immunoglobulin levels and higher B cell numbers than males.^24^ In addition, differential gene expressions between the B cells of females and males have been found.^25^

The survival benefit in female cancer patients may be supported by endocrine‐related reasons. It was reported that androgen receptors increased risk of tumor recurrence and reduced survival in liver cancer.^10^ Others have demonstrated that 2‐methoxyestradiol was able to induce apoptosis as well as autophagy, and 2‐methoxyestradiol could be considered as a promising tool against colorectal cancer.^5^ Prospective data indicate that estrogen may mediate some protection from cancer mortality in colorectal cancer.^26^, ^27^, ^28^

Our findings have several potential implications for future research and clinical practice. The first implication is that gender should be taken into consideration in the assessment of cancer‐specific survival. Future research should focus on improving the effectiveness of treatment in male cancer patients and perhaps explore different treatment strategies between male and female cancer patients. Because the results showed significant disparity in the cancer prognosis between male and female patients, gender as an important variable should be taken into consideration in clinical trial design.

Our study also had some limitations. Firstly, although SEER database contained data from 18 population‐based cancer centers in America, these results might not be extend to other areas. Secondly, differences in lifestyle, genetic test information, and access to care between male and female could not be included in this analysis due to missing data. Thirdly, although therapy type (whether patients underwent chemotherapy, radiotherapy, and surgery or not) was included in the adjusted model to decrease potential bias, the adjustment of the detailed information on treatment regimens were not conducted, because chemotherapy regimens and radiotherapy dose were unavailable in the SEER database. Finally, cause‐specific survival relied upon the accuracy of cause of death information in death certificates, and an alternative was to use relative survival based on population life tables matched on age, sex, and race.

In summary, this large‐scale population‐based analysis revealed that gender was a robust determinant of cancer‐specific survival, and female patient was associated with better prognosis. In consideration of the type of primary cancer, women showed survival benefit in lung, liver, pancreatic, colorectal, stomach, and esophageal cancer, and men showed better survival in bladder cancer. Except for kidney cancer, gender disparity in cancer prognosis was consistent between M0 and M1 disease.

## 4 MATERIALS AND METHODS

### 4.1 Patients and data collection

SEER database was used to identify eligible patients by SEER *Stat software (www.seer.cancer.gov/seerstat) version 8.3.6, which contains data from 18 population‐based cancer centers in USA.^19^ The most prevalent cancers (lung, esophageal, liver, pancreatic, stomach, colorectal, kidney, and bladder cancer) were included in this analysis. Patients coded with the primary site “lung,” “esophagus,” “liver,” “pancreas,” “stomach,” “colorectal,” “kidney,” and “bladder” were extracted from 1975 to 2016. Clinical stage was a key role for cancer prognosis, and SEER database used staging system of the 6th edition of the American Joint Committee on Cancer (AJCC) from 2004 to 2015. Thus, the 6th edition of the AJCC staging system was used in this study. Patients were excluded if they had multiple primary cancers.

### 4.2 Statistical analyses

Gender disparity of cancer prognosis was compared in all eligible patients and further stratified according to primary cancer type. Patients were divided into nonmetastatic (M0) and metastatic (M1) group. The primary outcome was cancer‐specific survival (CSS), defined as months from cancer diagnosis to cancer cause of death or to last follow‐up. Cause‐specific death as specified in SEER*STAT was used. Patients who died due to other causes were treated as censored at their death time, but patients with missing/unknown cause of death are excluded (<1% of overall cancer patients).^29^ We compared differences in CSS between men and women by the Kaplan–Meier method. Cox proportional hazards regression model was used to estimate HR and 95% confidence intervals (95% CI) for CSS. To reduce potential bias caused by confounding factors, firstly, we conducted analyses based on the year of diagnosis as a base model (model 1). Then, demographic, clinical, and treatment factors were added sequentially to establish adjustment models as model 2, model 3, and model 4. The detailed factors were as follow: (1) demographic factors including age, race, and marital status at diagnosis; (2) clinical factors including histologic type, histologic grade, and clinical stage; and (3) treatment factors including patients whether accepted chemotherapy, radiotherapy, and surgery. Furthermore, propensity score matching (a ratio of 1:1) was used to reduce potential bias caused by confounding factors. Data were analyzed using R software (version 4.0.3, Vienna, Austria). Statistical analyses were based on two‐tailed analyses with significance levels set at *p* < 0.05.

## AUTHOR CONTRIBUTIONS

*Study concept and design*: Yan He, Yu Zhang, and Xingchen Peng. *Acquisition and interpretation of the data*: Yan He, Yonglin Su, and Junsong Zeng. *Statistical analysis*: Yan He, Junsong Zeng, Yonglin Su, Xiaolin Hu, and Yu Zhang. *Drafting of the manuscript*: Yan He, Weelic Chong, Yu Zhang, and Xingchen Peng. All authors read and approved the final manuscript.

## CONFLICT OF INTEREST

The authors declare that there is no conflict of interest.

## ETHICS STATEMENT

Because the SEER database is a public database without identified information, the ethical approval for this study was waived by the ethics commission of the West China Hospital of Sichuan University.

## Supporting information

Supporting Information

## Data Availability

Data files can be downloaded directly from the SEER website (www.seer.cancer.gov/seerstat).
