# Supplementary material for: Cancer‐specific survival after diagnosis in men versus women: A pan‐cancer analysis
Source: MedComm (2020). 2022 Jun 30;3(3):e145. doi: 10.1002/mco2.145 (PMC9246337; doi:10.1002/mco2.145)

Cancer-specific survival after diagnosis in men versus women: a pan-cancer analysis

Running title: Cancer-specific survival in men versus women

Yan He[1*], Yonglin Su [2*], Junsong Zeng [1*], Weelic Chong[3], Xiaolin Hu [4#], Yu Zhang[5#], Xingchen Peng[1#]

[1] Department of Biotherapy, West China Hospital, Sichuan University, Chengdu, Sichuan, China.

[2] Department of Rehabilitation, West China Hospital, Sichuan University, Chengdu, China.

[3] Department of Medical Oncology, Thomas Jefferson University, Philadelphia, PA, USA.

[4] Department of Nursing, West China Hospital, Sichuan University, Chengdu, China

[5] Affiliated Hospital of Chengdu University, Chengdu, Sichuan, China

*These authors contributed equally to this work.

#Correspondence to: Xingchen Peng, email: pxx2014@163.com; Yu Zhang， email: tnt1057@outlook.com; Xiaolin Hu, email: huxiaolin1220@126.com.

Table S1. Cancer-specific survival in men versus women after propensity score matching.

Cancer types	Male		Female		HR (95%) of CSS
	Events	Total patients	Events	Total patients	
Total patients	111776	233820	101096	233820	1.203 (1.192-1.213)
Lung cancer	44791	65044	41882	65044	1.214 (1.198-1.23)
Esophagus cancer	4513	6122	4370	6122	1.07 (1.026-1.116)
Liver cancer	2334	3712	2235	3712	1.11 (1.047- 1.176)
Pancreatic cancer	8604	10576	8458	10576	1.11 (1.077-1.144)
Stomach cancer	5679	8512	5724	8512	1.032 (1.005-1.07)
Colorectal cancer	29543	88680	27019	88680	1.136 (1.118- 1.155)
Kidney cancer	5194	25318	4446	25318	1.186 (1.14-1.235)
Bladder cancer	5931	22242	5536	22242	1.141 (1.1-1.183)

Figure S1. Kaplan-Meier curve in non-metastatic lung (A), esophageal (B), liver (C) and pancreatic cancer (D).

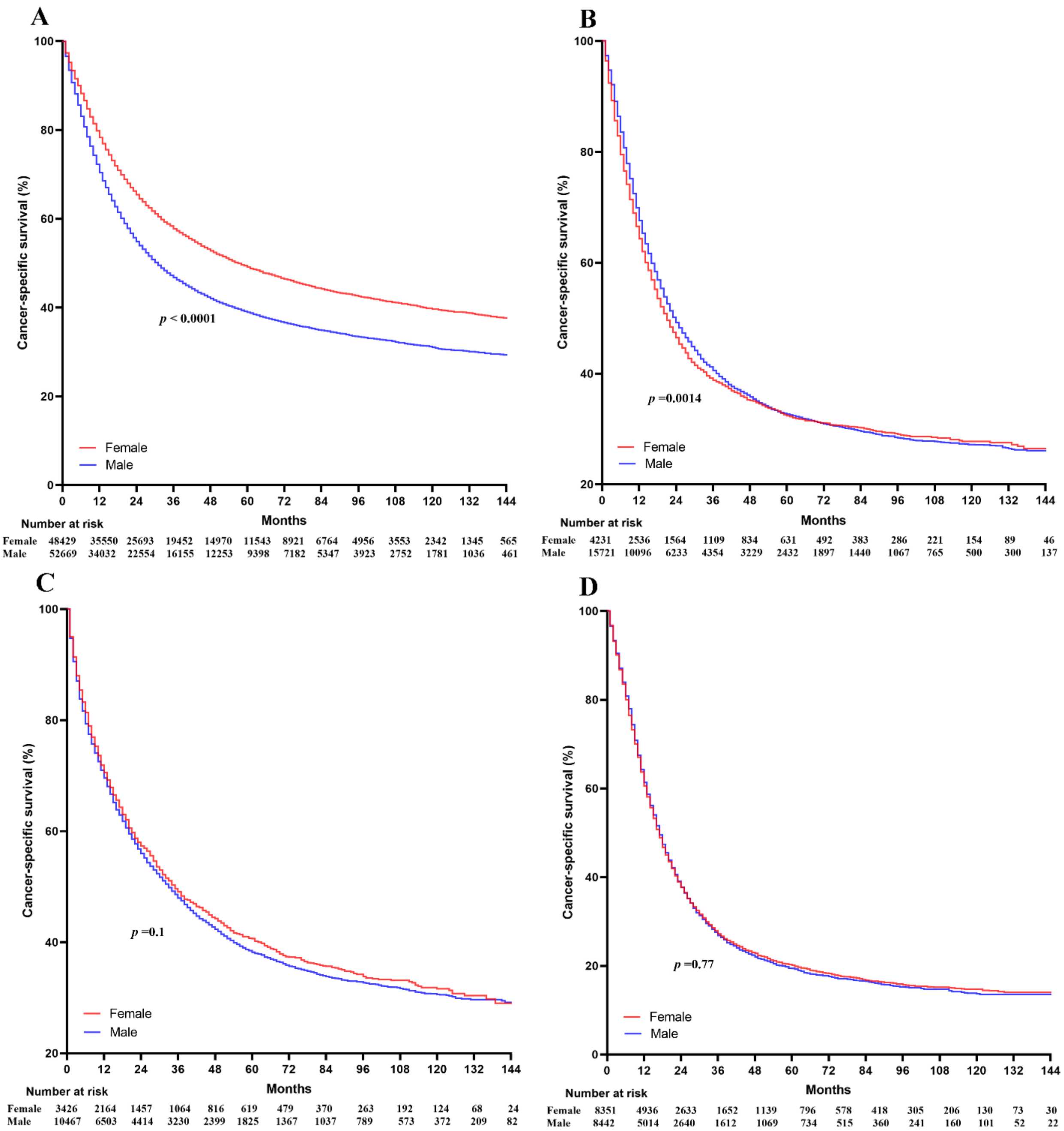

Figure S2. Kaplan-Meier curve in non-metastatic stomach (A), colorectal (B), kidney (C) and bladder cancer (D).

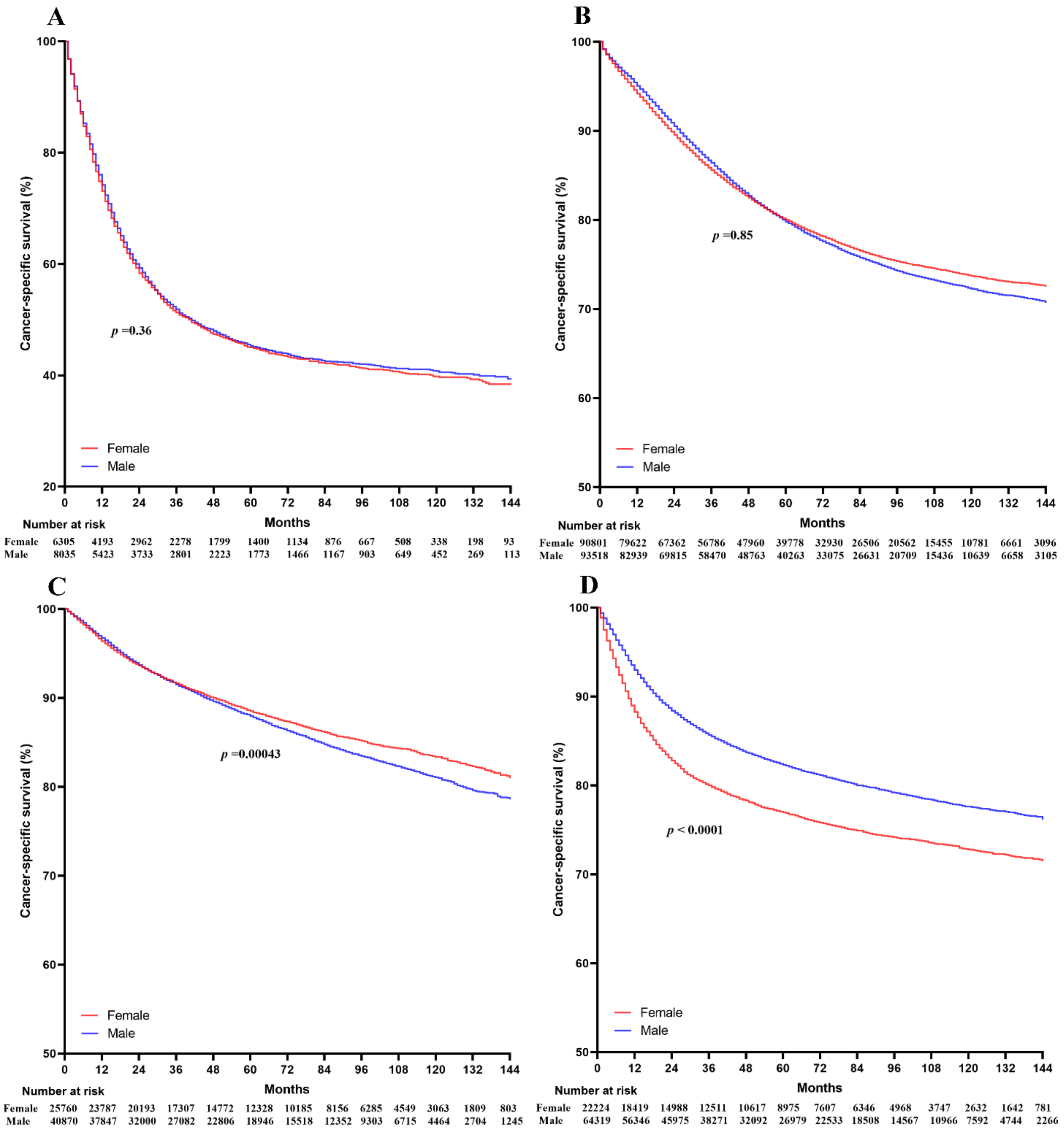

Figure S3. Kaplan-Meier curve in metastatic lung (A), esophageal (B), liver (C) and pancreatic (D).

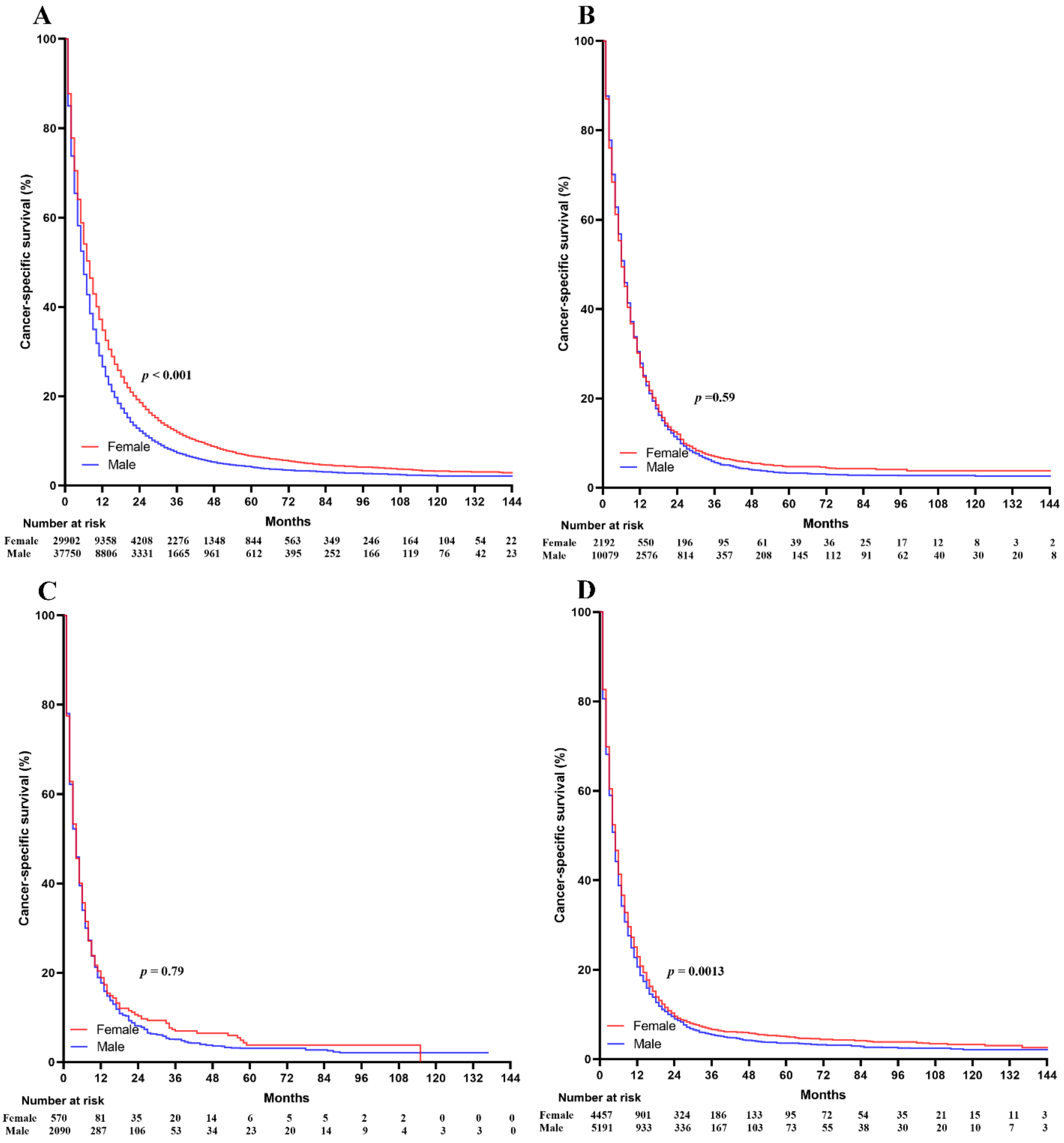

Figure S4. Kaplan-Meier curve in metastatic stomach (A), colorectal (B), kidney (C) and bladder cancer (D).

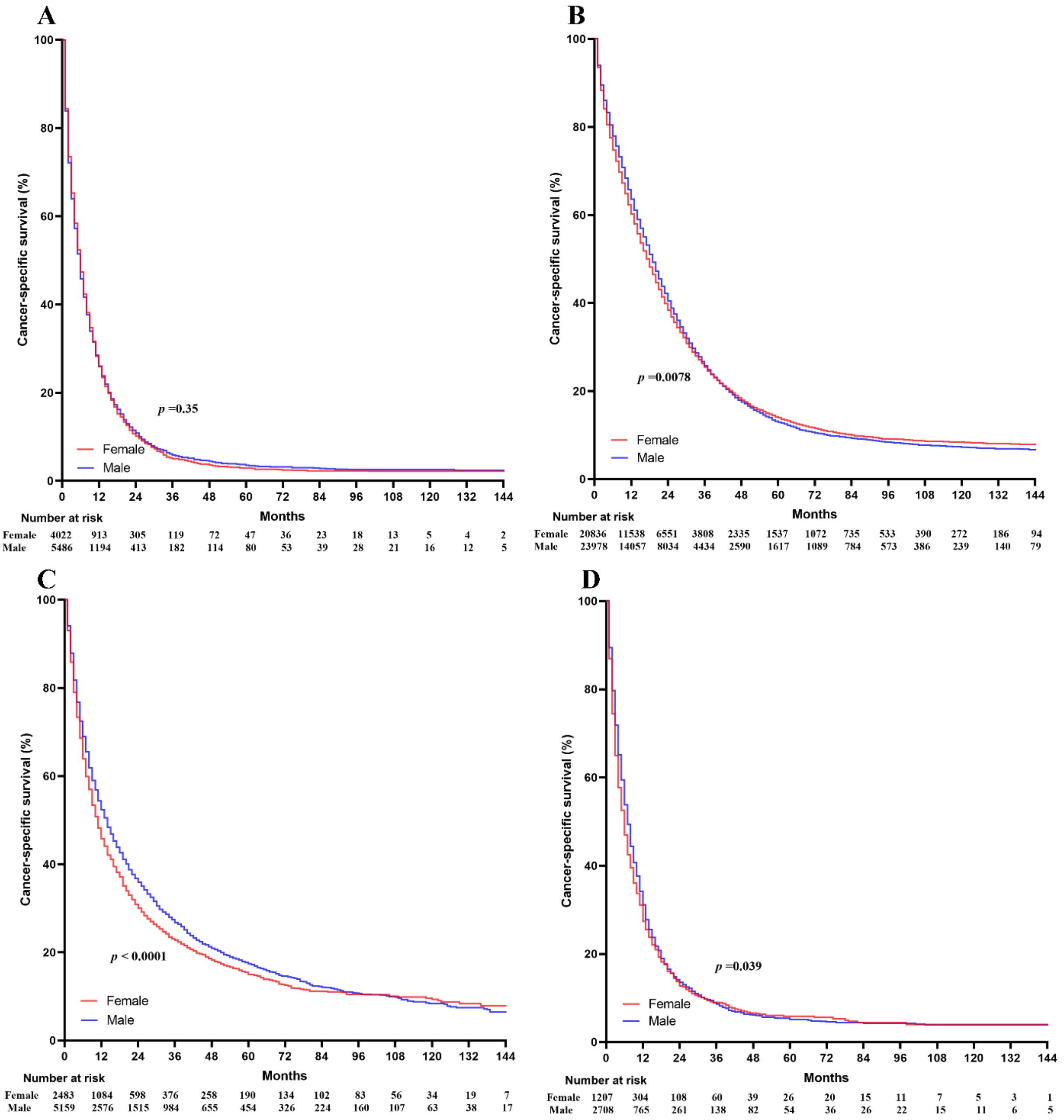

Supplement: Supplementary file 1 — Supporting Information [file MCO2-3-e145-s001.pdf]
